# Personalized Health Care System with Virtual Reality Rehabilitation and Appropriate Information for Seniors

**DOI:** 10.3390/s120505502

**Published:** 2012-04-30

**Authors:** Diego Gachet Páez, Fernando Aparicio, Manuel de Buenaga, Víctor Padrón

**Affiliations:** Escuela Politécnica, Universidad Europea de Madrid, Villaviciosa de Odón 28670, Spain; E-Mails: fernando.aparicio@uem.es (F.A.); buenaga@uem.es (M.B.); victor.padron@uem.es (V.P.)

**Keywords:** elders' health care, information access, wellbeing, ambient assisted living, disability

## Abstract

The concept of the information society is now a common one, as opposed to the industrial society that dominated the economy during the last years. It is assumed that all sectors should have access to information and reap its benefits. Elderly people are, in this respect, a major challenge, due to their lack of interest in technological progress and their lack of knowledge regarding the potential benefits that information society technologies might have on their lives. The Naviga Project (An Open and Adaptable Platform for the Elderly and Persons with Disability to Access the Information Society) is a European effort, whose main goal is to design and develop a technological platform allowing elder people and persons with disability to access the internet and the information society. Naviga also allows the creation of services targeted to social networks, mind training and personalized health care. In this paper we focus on the health care and information services designed on the project, the technological platform developed and details of two representative elements, the virtual reality hand rehabilitation and the health information intelligent system.

## 1. Introduction

**Figure 1.**
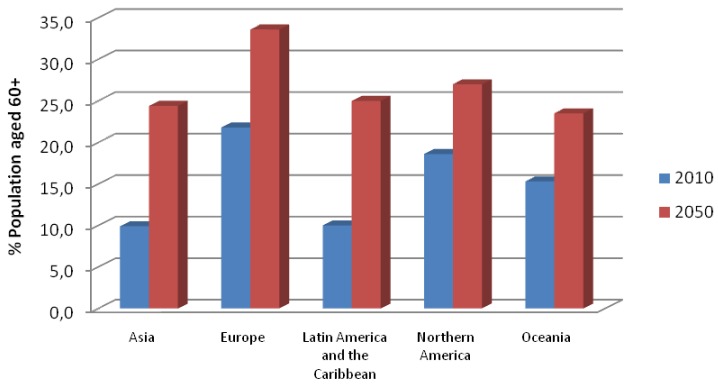
Demographic change according to the foresight of the United Nations. Available online: http://esa.un.org/unpd/wpp/index.htm (accessed on 21 May 2011).

Today, developed countries have great difficulties with effective health services and quality of care in a context marked by the population ageing. This general world trend, that can be seen in Figure 1, has dramatic effects on both public and private health systems, as well as on emergency medical services, mainly due to an increase in costs and a higher demand for more and improved benefits for users, as well as for increased personal mobility. This demographic change will lead to significant and interrelated modifications in the health care sector and technologies promoting independence for the elderly.

**Figure 2.**
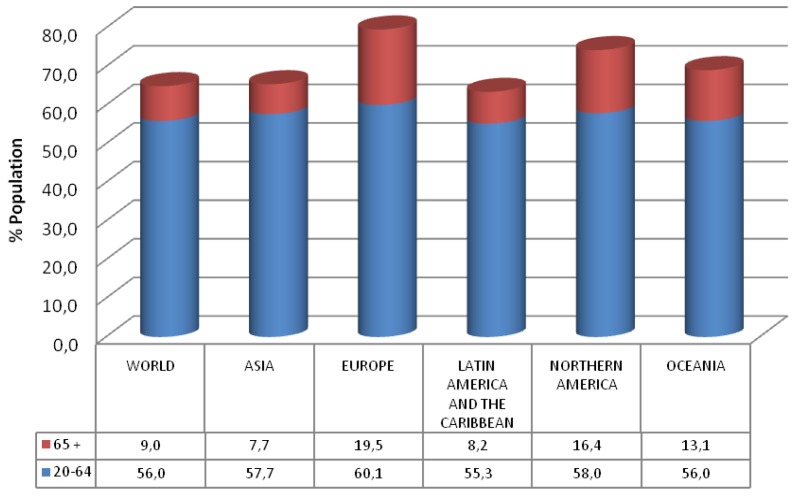
United Nations, Department of Economic and Social Affairs, Population Division (2011). World Population Prospects.

From Figure 2, as representative data approximately 60% of the European population (58% in Northern America) is made up of people aged 20 to 64 years, while the 65 and over group covers 19% (16% in Northern America). Thus, there are 3–4 working employees for every pensioner. On the other hand, it is estimated that the 20 to 64 years old group will decrease to 55% and the over 65 group will increase to 28% by the year 2050, making the proportion 1 to 2 instead of 1 to 3–4. Spending on pensions, health and long-term care is expected to increase by 4–8% of the GDP in the coming decades, with total expenditures tripling by 2050.

People live longer in developed countries as a result of better living and health conditions. For example, in North America only 4.5% of population over 65 years old lives in nursing homes, a percentage that has decreased in recent years. The elder population is increasingly prepared for aging well due to a decrease in disability, resulting in the elderly being more active in their daily lives. Despite the improvement in conditions for coping with ageing and an increasingly active lifestyle, there are obvious changes that occur in behaviors and skills during the latter part of life.

These changes may include decreases in social relations and physical abilities, loss of memory, comprehensive and cognitive functions. Previous studies have shown that the ageing process is accompanied by a decrease in neuromotor and cognitive functions. Compared to young people, the elderly demonstrate poor performance on tests, including reaction times, motor coordination, short-term and complex or abstract conceptualization. In general, these changes result in a decline in the quality of life.

Another important impact that can be seen particularly in persons living in nursing homes is boredom. Participation in social activities does not necessarily improve this feeling and sometimes creates negative attitudes in participants, although activities based on individual preferences can have positive effects and help to overcome boredom, increasing the quality of life for elderly. It is a challenge to find innovative activities that involve the aged and encourage them to keep on practicing with the activity.

An adequate understanding of the disuse of motor and cognitive functions can help to prevent the decline in these skills and participation in activities based on individual preferences can reduce boredom. There is a real need for activities that address these two concepts, and these activities may be none other than for example mental exercises and social networks specially designed for seniors.

The current trend is to improve the quality of life of elderly people not only extending the lifetime. The “gerontechnology”, see for example [1], is a very active discipline focused on improving the lives of elders, considered as a special group of users whose particular skills and needs in social and cognitive levels should be taken into account during the design process of any technology solution focused on this group. We must also consider that older people often do not feel comfortable in handling a computer and the use of technological devices seems complicated for them. This problem may be worse considering the decline in cognitive, visual or motor abilities.

The Naviga project (2009–2012) is an European initiative funded by the Eurostar R&D program, whose main objective is to provide these collective tools, devices and methods to enjoy personal autonomy and a better quality of life, to do that, within the project we are developing an integrated technological platform to provide for example Internet access through a computer or TV.

In addition, the proposed platform will facilitate the incorporation of elders and people with different functional capacity to the Information Society, through the use of special input devices, an adaptable Web Navigator, social networks, applications to improve the cognitive ability (serious games) and personalized health services.

The consortium comprises five SMEs conducting research (investment min. 20% of annual turnover in R & D), a university and two end users are also involved in the project (an hospital and a daily care health centre located in Madrid area).

## 2. Objectives

The Naviga project, through the use of information and communication technologies, intended to cover a range of social and health objectives aimed to improve access to the Information Society by the elderly and people with disabilities. Within Naviga we will develop an open platform and adaptive technology for various purposes detailed in the following subsections.

### 2.1. Technological Oriented Objectives

As Technological oriented objectives we can mention the following:

- Development of an adaptive communication interface between user and computer or television, facilitating the understanding of Internet and new technologies to people with a low-tech profile, while encouraging its use by providing a simple and friendly human machine interface.
- Integration with different support products on the market to ensure that users can use those techniques.
- A platform development that allows rapid creation of services and applications specifically designed for the elderly and disabled people with a common API.

### 2.2. Social Oriented Objectives

In this case, the main social objectives lie on the attempt to bridge the gap that prevents the elderly and people with disabilities access the Information Society. To do this, we are developing simple mechanisms for interaction between technical elements (computer, television or special input devices in place of keyboard) and people like, for example, an accessible Web browser to improve usability through the use of alternative hardware to keyboard or voice commands. The browser will be compatible with common support and aid products for elder people. We are also developing a social network among people with the same disability, where users can find people with common interests and concerns, as well as share information, experiences and advice.

### 2.3. Health Oriented Objectives

Similarly, the Project will provide a range of health-oriented goals that help elderly remain active through mental training exercises and, otherwise, assist medical staff in the task of monitoring the treatment of these people from homes, through services and games that allow mental training (mind training), suggesting exercises to keep the mind active and getting people to communicate and participate to a greater extent in their social community. This will prevent premature degeneration of mental activity, and improves the senior's mood with functional diversity by increasing the feeling of being useful to society around them.

Although little is known about the perceived benefits of mental games for the elderly [2], there is a small but growing body of research evidence in support of the notion that brain exercises can have a significant positive impact on the elderly's mental and physical health and wellbeing [3], as for example a better information processing, reading, comprehension, memory, self-image, *etc.*

Development of personalized health services is also a part of the Naviga's objectives, such as warning and reminder system for medication adherence through an automatic smart pill dispenser or home rehabilitation physiotherapy through virtual reality applications. In the last case, the main goal is to recover the functionality of the hand of patients using a glove that makes measurements of the angles of each phalanx up to 22 degrees of freedom with high accuracy. The device uses a strain sensing technology that transforms the movement of the hand and fingers to digital data in real time. In medicine Virtual Reality is employed for surgical simulation training (e.g., [4]), also, it is a common element in ambient Intelligence systems [5] and in other areas as three-dimensional anatomy (e.g., [6,7]), surgery planning (e.g., [8]) and the treatment of anxiety disorders (e.g., [9]). Virtual reality is a very attractive environment for the treatment of phobias because of its safety.

The project has also included a module for personalized access to health information in the Internet. Some components of this development have been made following MedicalMiner project objectives, that can be found in [10], among which can be highlighted: (1) development of new techniques of data mining focus on the analysis of personal health information; and (2) design of an intelligent medical information system capable of text and data mining. Specifically, the module implemented for the Naviga platform provides direct access to selected health information sources such as MedlinePlus, [11], including automatic text content analysis based on Gazetteer module of General Architecture for Text Engineering software (GATE, [12]) and medical terms covered by the Open Biomedical Annotator (OBA, [13]) and Freebase ([14–16]).

## 3. Technological Platform's Architecture

Among the initial services of the platform, there are technical difficulties related to the application area. For example, the development of an accessible Web browser must be multimodal and interoperable in order to take into account the needs of all members of the group, which greatly complicates the solution due the diversity of users. Also, the use they make of the social network can be very different, both, use objectives (social relationships, share experiences, recommend support products) and access to services, must provide simple user interfaces, easy to use and highly adaptability to the preferences and characteristics of each person. Our platform allows integrating the specific elements of all the targeted users of the project.

**Figure 3.**
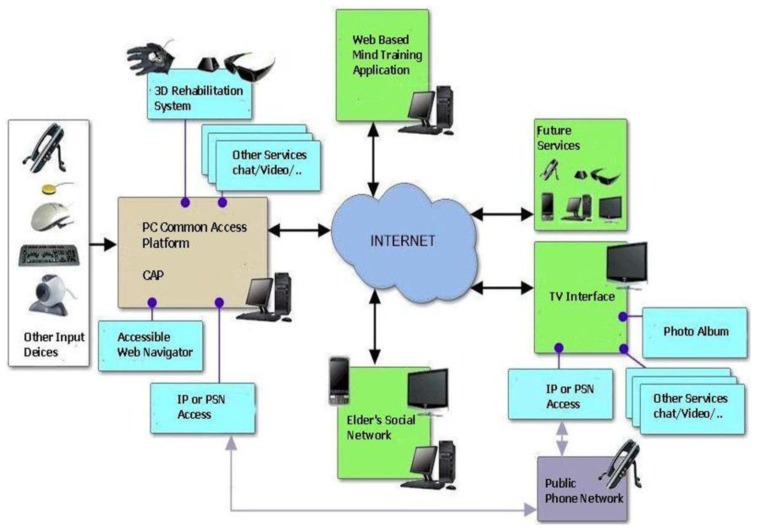
Naviga's Technology Platform.

The Naviga platform provides an open system based on SOA (Service Oriented Architecture) that enables and facilitates the development of new applications and services that seamlessly integrate with existing modules without need of an expert knowledge of the lower layers architectures and languages. Also, open source implementation based on Java EE and scripting languages like JavaScript, and compliance with accessibility standards of the ISO and the recommendations of the WAI, ensures continuity of service and support the development of the platform. The technology platform we are developing within the Naviga Project, depicted in Figure 3, must solve two major technical challenges:

Firstly, the connection to the platform in an interoperable way of different support products and communication interfaces, integrating health monitoring devices that generate medical alerts, fall detection systems and security alarms, and devices that enable accessibility to users with motor or cognitive disabilities to information and entertainment services. The number of support products available in the market is very high, but often not compatible with each other or have the same degree of utility to different users who share a disability. It is therefore necessary to develop a common multi-modal interface that simplifies the integration between computers and any specific support product. It should also be kept in mind the need for multi-channel access, granting Internet navigation through the computer, television or mobile devices.

**Figure 4.**
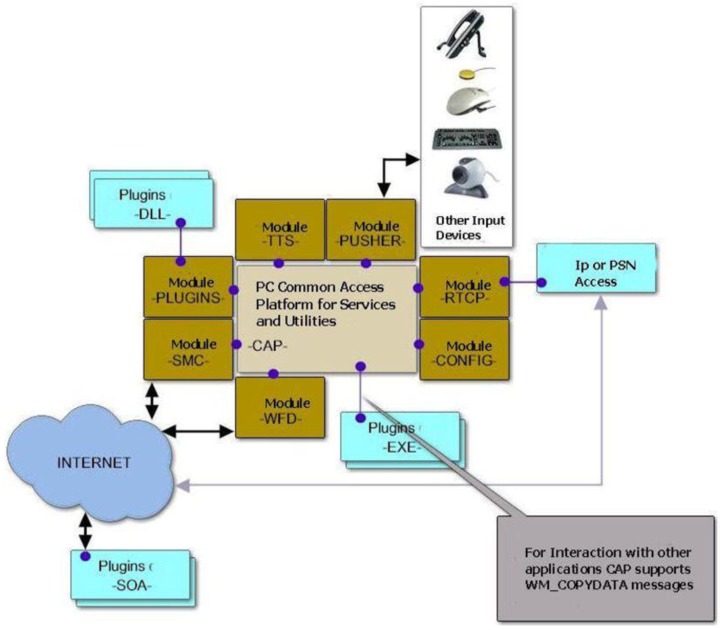
A detailed High Level View of the Common Access Platform.

Second, the development of a set of tools to create and deploy services and applications to guarantee compatibility and rapid integration of new services and devices on the platform, while providing a common adaptive and easy to customize interface for user interaction, that is the function of Common Access Platform (CAP), pictured in Figure 4, a shell running in place of the Microsoft Windows Operating System that implements several modules as for example: Short Message Commands (SMC), Web File Download (WFD), Text to Speech Conversion (TTS) and all elements to manage future applications and services to be included in the platform.

The main module inside the CAP is the so called “PANORAMA” which implements the user GUI and has a similar behavior to the Windows desktop, but with some innovative characteristics that allow meets the specific 65+ user requirements (size of icons, colors, *etc.*) and including likewise key aspects of advanced user interfaces.

A special feature of the CAP is that it must be able to speak and perform voiceovers at the request of any other module or plug-in in the platform. This ability to speak is performed by TTS module (Text to Speech), which will be able to interpret instructions to carry out a phrase, change of speaker or speed, volume, *etc.* This module is capable of aloud the name of the icon over which the user is, or allow the CAP to alert user of events or errors via voice messages. This means that any module, plug-in or Naviga service must be able to communicate with the user using phrases.

## 4. Virtual Reality Hand Rehabilitation. An Example of Naviga's Health Service

Virtual Reality (VR) is a human-computer interface technology that allows the user to experience and interact with virtual environments. The three-dimensional graphics and the possibility to interact with the environment give the participant the feeling that he is part of this world: immersion is the professional term for this. Stereo sound may contribute to the immersion, as well.

Virtual Reality is used in medicine for surgical simulation training, three-dimensional anatomy and surgery planning. Also Virtual Reality is successfully used in the treatment of phobias. Virtual environments are safe, controlled environments and therefore attractive environments for the treatment of phobias. Besides the feeling of safety the patient has to experience the virtual environment as real and has to feel a part of it in order to conquer the fear. From these studies it is concluded that it is possible to create a virtual environment that it is experienced as real and immersive. After the successful treatments of phobias with Virtual Reality more and more interest arises for Virtual Reality in cognitive and physical rehabilitation, see for example [17].

In the framework of the Naviga project, we have chosen a health service for hand rehabilitation based on Virtual Reality for the improvement of the patient's outcome. This service is fully integrated within Naviga Platform allowing users to do rehabilitation exercises at home. In the current state of the project, the VR hand rehabilitation system has been already implemented. We have designed its function in coordination with doctors of a Madrid area Hospital and we'll evaluate the results with a set of users in a next phase.

Subjects taken part on the rehabilitation therapy are mainly patients who had a traumatic hand injury or surgery due to a disease. Patients after brain injury and with cognitive and mobility handicap are not often taking part of this rehabilitation sessions, although sometimes they are included and can represent also a challenging task for the therapists. Rehabilitation sessions are based on exercises involving actions related to an object:

- Reaching objects at different heights and positions.
- Grasping objects of different shapes and sizes.
- Functional manipulation of objects.

The service is based on a CyberGlove^®^ equipment. This data glove is a lightweight, comfortable, fully instrumented glove that provides up to 22 high-accuracy joint-angle measurements. It uses proprietary resistive bend sensing technology to transform, with high accuracy, hand and finger motions into real-time digital joint-angle data. An option to the glove, the CyberTouch system provides vibrotactile feedback to the fingers and palm.

**Figure 5.**
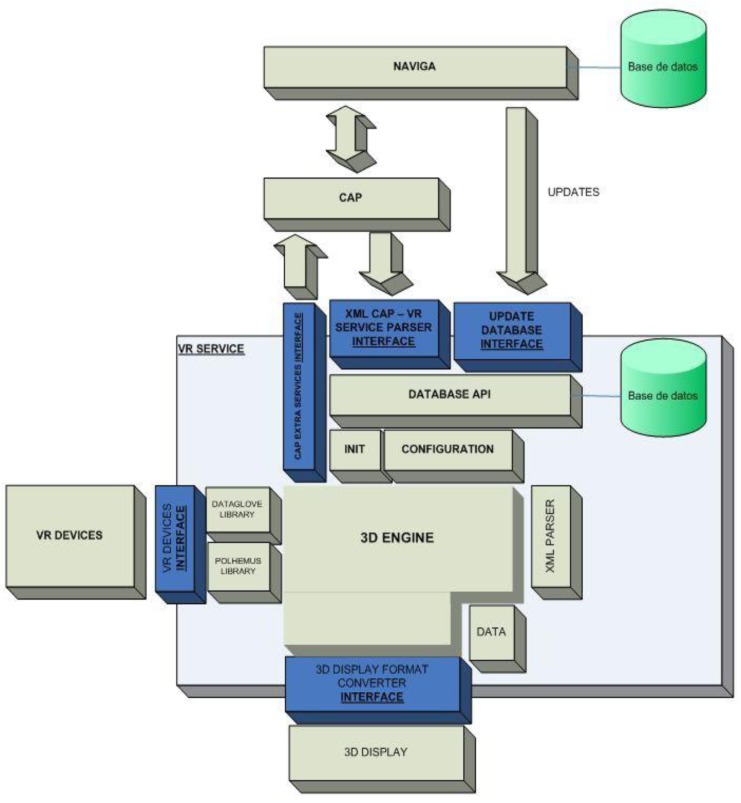
Naviga—VR-Hand-Rehabilitation Service. Blue blocks represent the interfaces involved in this service.

The VR-Hand Rehabilitation Service enclosed within Naviga can be defined as observed in Figure 5. As we can note from the diagram, the interaction between Naviga and the service is mainly through the CAP, being important to remark that the interface between CAP and the service is designed as lying on an xml interface. This means, every module in the service is launched trough a defined xml file, but in general all modules interact with the local database. The VR-Service will take profit of other CAP Services (TTS, Video Player, *etc.*) as well.

The software developed within the VR-Hand-Rehabilitation service it composed of the following modules:

- Module 1. Database Configuration of the system.
- Module 2. DataGlove Initialization.
- Module 3. DataGlove Calibration.
- Module 4. DataGlove Re-Calibration.
- Module 5. Real-Time VR System Application.
- Module 6. Offline Session Reconstruction.

**Figure 6.**
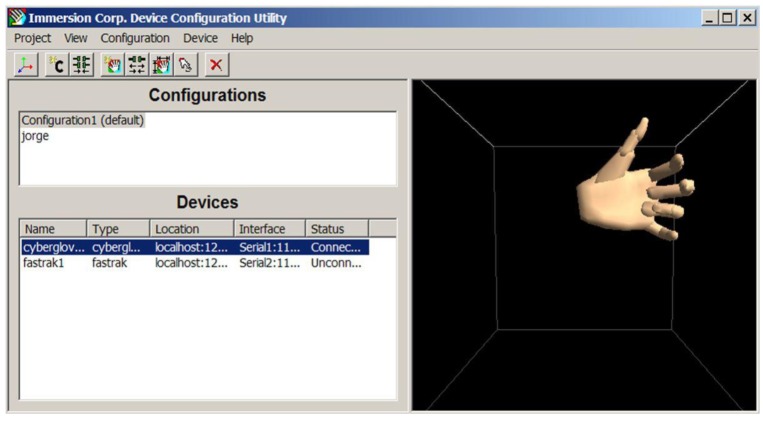
Data Glove Initialization subsystem.

These modules cover from the Initial Local Database and DataGlove Configuration to the execution of a rehabilitation session and further analysis. Figure 6 shows the DataGlove Initialization module.

## 5. Intelligent Recognition of Proper Health Information for Seniors

**Figure 7.**
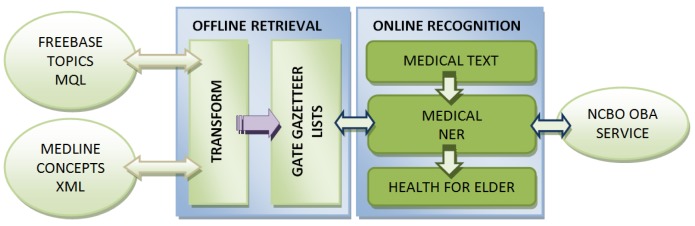
Health information service for elderly architecture.

The intelligent systems group at Universidad Europea de Madrid has carried out a notable effort building and testing a medical named entity recognition system over clinical and medical reports, using two main stages: first, an offline medical terms retrieval and, two, an online recognition over a text entered by the user. These high level processes are sketched in Figure 7. The following sections describe these offline and online phases, as well as the main results of the assessment conducted.

### 5.1. Offline Retrieval

The proposed system pre-processes a set of medical terms compiled into lists, which are then used by the Gazetteer component of the ANNIE system included in the GATE distribution. To build these lists of medical terms we have preprocessed data from Freebase, using the MQL service [18], and MedlinePlus, downloading automatically an XML file provided in [19].

Freebase structures the content based on the called topics, types, domains and properties. The topics group descriptive information associated with a named entity (we use the name to the NER online task) and they keep a semantic relation with the types and properties: The topic “is_a” type or “has_a” property. The types are grouped into domains (e.g., medicine, travel, *etc.*) to which are assigned an identifier associated with the link to the content (/medicine, /travel, *etc.*) and contain a set of properties. The domain of interest in our case is medicine and to decide the best types we just proceed revising the semantic information of the types with the highest number of instances, considering merely data of interest to this research and taking into account the relationship between topics and properties “the topic has_a property”. The starting point for the selection of types is as follows. It can be noticed how even though any of these seven types may be of interest for a labeling to improve the understanding of a clinical case, just Disease or medical condition, Medical treatment and Symptom have a circular relationship, meaning that they include relations through properties whose information is filtered from the other two types: Treatment and Symptom properties of the first are extracted from the second and third respectively, Side effect and Used to treat properties of the second are extracted from the third and the first respectively, Symptom of and Side effect of properties of the third are extracted from the first and the second respectively. This circular relationship between types and properties implies two main considerations: (1) all semantic content could be centralized through the developed system, without linking external content at all; and (2) the final user only needs to handle a limited number of relationships. These two considerations (involving a significant simplification of the total) together with the existence of the type Risk Factor (making possible the identification of types in which old age is a risk factor), provide a method to locate health information of particular interest to elder people.

**Figure 8.**
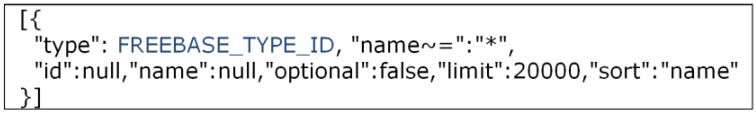
MQL offline query pattern.

Table 1 shows the relations referred above with the Freebase identifier needed to prepare the query. To obtain the topic's names belonging to the types Disease or medical condition, Treatment or Symptom, MQL generic queries have been built as it is shown in Figure 8. The limit is set to a greater value than that of the type which has more topics, consequently the totality of them is got, and has been added a clause to sort them alphabetically.

Freebase has also been employed in other areas, such as the works in the context of integration of knowledge, e.g., in [20,21], or as a component to improve enterprise data analitics, e.g., [22]. Another source of medical terms that has been preprocessed is MedlinePlus, using the XML vocabulary file provided, as it was mentioned above. MedlinePlus is one of the National Library of Medicine (NLM, [23]) resources offered to the public, a finder of health problems with up to date and easy to read information about nearly 900 health topics (with other media contents) and 37 million visitors [24].

We have extracted the concepts tagged as “MedicalTopicName” for the two available languages in the XML, English and Spanish. Other information included in these files (one for each language) are the classification groups found within the contents of MedlinePlus, among which is the Seniors group that enables the system to discern the issues of interest to older people.

### 5.2. Online Recognition

Online processing phase mainly consists in the recognition of entities (previously stored in lists) on medical texts, showing supporting information on those that are of concern for the seniors in a suitable format and language.

**Figure 9.**
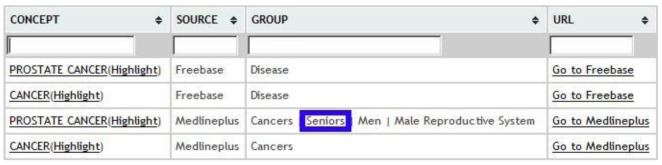
Relevant entity recognition from MedlinePlus for seniors.

**Figure 10.**
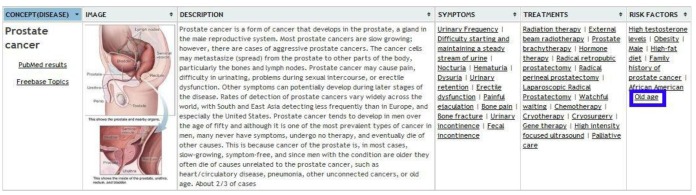
Relevant entity recognition from Freebase for seniors.

We have developed a Web user interface and an XML service. As an example, assuming an input text containing the term “prostate cancer”, we would have the option to filter the content for both entries from MedlinePlus to those from Freebase, marked on blue in Figures 9 and 10 respectively. It is possible to perform annotation via OBA service ontologies, although currently the only one that is fully integrated is Medlineplus Health Topics.

Integration of this module with the Naviga CAP through SOA provides an intelligent mechanism for the recognition of medical concepts especially relevant for older people, for whom it might be interesting to have their own medical history stored into the platform.

### 5.3. System Evaluation

An evaluation of this module has been carried out with very positive results [25]. The assessment was conducted on users in a different field, for which the tool can be useful too. In particular, we have created two groups of students from second year of medical school, testing them on a clinical case [26]. The test was divided into an objective multiple choice questionnaire, consisting of 10 questions, and two Likert type subjective questionnaires. In one group, consisting of 26 students, was allowed to use internet searches to solve the objective examination, while the other was provided access to perform the same task but making use of the module through our Web interface only. Students in this latter group have answered the two Likert type questionnaires mentioned before, regarding usability and the contribution provided by the system about learning.

## 6. Conclusions and Expected Results

The Naviga Project (An Open and Adaptable Platform for the Elderly and Persons with Disability to Access the Information Society) has as goal the design and develop of a technological platform allowing elder people and persons with disability to access the Internet and the Information Society. In this paper we have focused on the health care and information services designed on the project, the technological platform developed, and details of a representative element already in function, the virtual reality hand rehabilitation. As mentioned above, from the point of view of development, the project's expected results are:

- A hardware interface device adaptable to all seniors and people with disabilities enabling the interaction with computer or television.
- A framework (tools and methods) to create and deploy services and applications.
- The development of services including a Web browser that allows access for elders and disabled people to the Internet.
- Two technology demonstrators in the field of e-Health and entertainment.
- An analysis of business opportunities and business requirements (identifying their strengths and weaknesses) for the successful commercialization of the project results.

During the running of Naviga project two case studies/scenarios are being implemented to demonstrate the functionality of the framework developed. One dealing with rehabilitation at home based on virtual reality, while another scenario will be developed and evaluated in a care centre for elders and people with disabilities aiming their access to the Information Society through the adaptable Web browser and in particular social networks and mental training. The scenarios will have real participation of end users to validate the technological advances.

## Figures and Tables

**Table 1.** Selected freebase types and properties.

**Freebase Types and Properties from Medical Domain**		

***Type***	***Properties***	***ID***

Disease or medical condition	Risk Factors	/medicine/risk_factor
	Symptoms	/medicine/symptom
	Treatments	/medicine/medical_treatment

Medical treatment	Side effects	/medicine/symptom
	Used To Treat	/medicine/disease

Symptom	Symptom of	/medicine/disease
	Side effect of	/medicine/medical_treatment

Risk factor	Diseases with this Risk Factor	/medicine/disease
